# Olfactory bulb encoding during learning under anesthesia

**DOI:** 10.3389/fnbeh.2014.00193

**Published:** 2014-06-05

**Authors:** Alister U. Nicol, Gabriela Sanchez-Andrade, Paloma Collado, Anne Segonds-Pichon, Keith M. Kendrick

**Affiliations:** ^1^Sub-department of Animal Behaviour, University of CambridgeCambridge, UK; ^2^Wellcome Trust Sanger InstituteHinxton, Cambridge, UK; ^3^Department of Psychobiology, Universidad Nacional Educación a Distancia (UNED)Madrid, Spain; ^4^Bioinformatics Group, The Babraham InstituteCambridge, UK; ^5^Key Laboratory for Neuroinformation, University of Electronic Science and Technology of ChinaChengdu, China

**Keywords:** anesthesia, microdialysis, mitral cells, multiarray electrophysiology, neurotransmitters, olfactory bulb, olfactory learning, social transmission of food preference

## Abstract

Neural plasticity changes within the olfactory bulb are important for olfactory learning, although how neural encoding changes support new associations with specific odors and whether they can be investigated under anesthesia, remain unclear. Using the social transmission of food preference olfactory learning paradigm in mice in conjunction with *in vivo* microdialysis sampling we have shown firstly that a learned preference for a scented food odor smelled on the breath of a demonstrator animal occurs under isofluorane anesthesia. Furthermore, subsequent exposure to this cued odor under anesthesia promotes the same pattern of increased release of glutamate and gamma-aminobutyric acid (GABA) in the olfactory bulb as previously found in conscious animals following olfactory learning, and evoked GABA release was positively correlated with the amount of scented food eaten. In a second experiment, multiarray (24 electrodes) electrophysiological recordings were made from olfactory bulb mitral cells under isofluorane anesthesia before, during and after a novel scented food odor was paired with carbon disulfide. Results showed significant increases in overall firing frequency to the cued-odor during and after learning and decreases in response to an uncued odor. Analysis of patterns of changes in individual neurons revealed that a substantial proportion (>50%) of them significantly changed their response profiles during and after learning with most of those previously inhibited becoming excited. A large number of cells exhibiting no response to the odors prior to learning were either excited or inhibited afterwards. With the uncued odor many previously responsive cells became unresponsive or inhibited. Learning associated changes only occurred in the posterior part of the olfactory bulb. Thus olfactory learning under anesthesia promotes extensive, but spatially distinct, changes in mitral cell networks to both cued and uncued odors as well as in evoked glutamate and GABA release.

## Introduction

A number of studies have provided evidence for the occurrence of associative learning under general anesthesia. Initial findings using auditory fear conditioning paradigms suggested that learning under anesthesia only occurred if epinephrine was given during the conditioning procedure (Weinberger et al., 1984; Gold et al., 1985). However, subsequent studies using both auditory and olfactory conditioning paradigms have provided evidence for learning under anesthesia without the necessity for epinephrine treatment. For both auditory and olfactory conditioning under anesthesia evidence for altered responses of neurons in the medial prefrontal cortex (Rosenkranz et al., 2003; Laviolette et al., 2005) and lateral amygdala has been reported (Rosenkranz and Grace, 2002; Rosenkranz et al., 2003; Fenton et al., 2013). All these studies have used paradigms where a CS+ is associated with foot-shock and effects of associations with positive reinforcers have not been investigated. Additionally there is evidence from *in vivo* neurotransmitter release, and localized pharmacological intervention and electrophysiological recording studies in both sheep (Kendrick et al., 1992, 1997) and mice (Wilson et al., 1987; Brennan et al., 1998), that plasticity changes occurring within primary sensory cortex, notably the olfactory bulb, are important for learning. However, whether similar changes occur in the olfactory bulb during learning under anesthesia is unknown.

Odor learning in mammals, under various paradigms, has been shown to be supported, to a considerable extent, by biochemical and physiological changes occurring in the mitral cell layer of the olfactory bulb. Learning-related elevations in extracellular levels of glutamate and gamma-aminobutyric acid (GABA), and an increase in the ratio of GABA relative to glutamate have been found in both sheep (Kendrick et al., 1992) and mice (Brennan et al., 1998) following olfactory learning. This suggests a mechanism involving reciprocal increases in both excitation and inhibition, where the relative impact on inhibitory activity is greater. Other reported extracellular changes include increased noradrenaline, nitric oxide and aspartate (Kendrick et al., 1997; Brennan et al., 1998). In the accessory olfactory bulb (AOB), an area associated with pheromonal perception and learning, neurochemical (Brennan et al., 1995) and electrophysiological (Binns and Brennan, 2005) effects consistent with such a mechanism have also been reported in relation to the Bruce effect in mice, whereby exposure to the odor of an unfamiliar male causes termination of pregnancy. Local field potential recordings in the AOB suggest selective inhibition of the familiar pheromone in the underlying recognition system. In the main olfactory system however odor learning can be associated with both increased and decreased responses of mitral cells (Wilson et al., 1987; Kendrick et al., 1992).

One of the most robust models of olfactory learning in rodents is the social transmission of food preference. Rodents such as mice and rats are generally neophobic with regard to novel foods, preferring to eat food items which are familiar to them. However, following social interaction with a conspecific “demonstrator” which has previously consumed a novel food, otherwise naïve “observer” animals subsequently show a preference for the same novel food by consuming more of that food than an alternative novel one (Galef and Wigmore, 1983; Valsecchi and Galef, 1989). Indeed, the acquired attractiveness of the novel food may be such that the consumption of this food initially exceeds the preceding consumption of the animals’ normal daily diet (Galef and Whiskin, 2000). The social transfer of food preference does not require direct physical contact between the demonstrator and observer animals since it is mediated by carbon disulfide (CS_2_; Galef et al., 1988), a metabolic by-product carried in the exhaled breath of rodents. Effective training of the observer may be accomplished using an anesthetized demonstrator (Galef and Wigmore, 1983; Valsecchi and Galef, 1989), or even replacing the demonstrator with an artificial surrogate, such as a wad of cotton wool carrying a novel food odor and a few drops of CS_2_ (Galef et al., 1988).

We have previously provided behavioral evidence that social transmission of food preference can occur under anesthesia in mice, using anesthetized demonstrators (Burne et al., 2010), although whether this involves neurochemical and electrophysiological changes in the olfactory bulb similar to those seen following learning in awake animals is unknown. In another study on responses of single neurons in the olfactory bulb of anesthetized mice test odors were mixed with the anesthetic gas and produced reliable mitral cell responses (Lin et al., 2005). In the present study we therefore used this same approach to investigate firstly *in vivo* neurochemical changes occurring post-learning in anesthetized mice in response to odors smelled on the breath of a demonstrator mouse. During the learning phase of the experiment both observer and demonstrator mice were anesthetized. In a second experiment multielectrode array (24 electrode) electrophysiological recordings were used to monitor the olfactory responses of neurons in the mitral cell layer of the olfactory bulb in isofluorane anesthetized mice both during and shortly after learning using a paradigm where animals were exposed to a novel (CS+) odor with CS_2_.

## Experimental procedures

### Animals

All procedures were conducted under licence in accordance with UK Home Office regulations (Animals (Scientific Procedures) Act, 1986).^1^

The subject animals were adult male mice (129svC57B6) which were bred in-house (Babraham Institute) and maintained to an age of 3–6 months before any experimental procedures. Mice were housed in same-sex groups of 2–5 animals in standard M3 cages, under temperature-controlled conditions in a 12 h light—12 h dark cycle (lights on at 07:00 h). All animals were handled and weighed on each of the 2 days before the start of experimentation.

### Preparation of food odors

Scented foods were prepared by introducing an additive to the normal diet in powdered form. The additives used were cumin (0.2%), ginger (1%), coriander (1.5%) and cocoa (2.0%), each obtained from a local retailer and stored in airtight containers. Odor samples were prepared by inserting a non-airtight capsule containing 2–3 g of powdered food into a polyvinyl fluoride gas sampling bag (Adtech Polymer Engineering, UK). All air was removed from the bag, which was then refilled with 1 L of nitrogen gas (odorless). Preparation of scented foods and food odors was done in a room separate from areas where experimental procedures were performed. Odor bags containing CS_2_ (∼800 ppm) were prepared by placing a 1 cm square of filter paper into an evacuated odor bag, adding 0.2 µL of CS_2_ using a 10 µl precision syringe (Hamilton Bonaduz AG, Switzerland) through a sealed injection site, then refilling the bag with 1 L of nitrogen gas. Samples of N_2_ gas were also prepared.

### Bevavior

#### Restricted feeding

**Figure 1:**
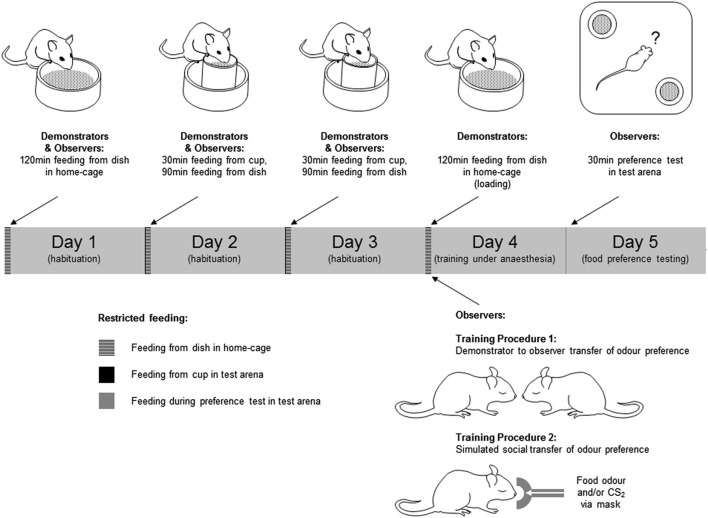
**Training and testing of mice in social transmission of food preference paradigms**. Behavioral procedures were conducted over a 5 day period. In the 3 days preceding training and testing, mice were submitted to a restricted feeding regime in which they were allowed free access to food in a fixed 2 h period each day. The food provided in this period was the normal daily diet in powdered form rather than the pellets to which they were accustomed. Access to drinking water was unrestricted during the restricted feeding regime. In days 2 and 3 of this regime the animals spent 30 min of the feeding period individually in the test arena (a 33 × 33 × 25 cm high white plastic container). In this period of habituation to the test environment, ∼2 g of the powdered food was provided in a feed cup—a plastic cylinder (4 cm diameter × 5 cm high) fixed in the center of a plastic dish (6 cm diameter × 2 cm high) to catch any spillage (Figure 1). On day 4, Demonstrator mice were “loaded” by being given unrestricted access to either a flavored food or the normal food, both in powdered form. Observer mice were anesthetized, and exposed to the training odor by one of two procedures; either (1) carried on the breath of an anesthetized demonstrator, or (2) introduced to the anesthetic gas with or without CS_2_. On day 5, mice were given a 30 min simultaneous choice test to determine their food preference. Mice which consumed none of either food in the preference test were excluded from further analysis.

Prior to training, mice were submitted to a restricted feeding regime (see Figure 1 and legend). During the restricted feeding regime, animals exhibiting weight loss exceeding 15% of initial weight were returned to normal feeding and excluded from further procedures.

#### Training procedure 1—social transfer of food preference

On day 4 of restricted feeding, demonstrator mice were transferred individually to a clean cage which was the same as the home-cage but with no sawdust, and allowed to feed for 2 h *ad libitum* from a dish (6 cm diameter × 2 cm high) containing 2–3 g of food. The food presented in this loading period was either the normal powdered diet, or food scented with either cumin or cocoa. Mice eating less than 0.1 g in this period were not used as demonstrators and were excluded from further experimental procedures. After loading, the demonstrator was anesthetized (25% Hypnorm, 25% Hypnovel, i.p., 0.1 ml/g body weight). Observer mice were anesthetized (1.5% isofluorane in O_2_ delivered at 150 ml/min via a mask over the nose) and, when fully anesthetized, placed individually in close nose to nose proximity, but without actual direct physical contact, with the demonstrator mouse for 1 min. There were two groups of trained observers: for one group the training food (i.e., the food used to load the demonstrator) was cocoa-scented (cocoa-trained, *n* = 10 mice), for another group the training food was cumin-scented (cumin-trained, *n* = 7). For a third group the demonstrator had been fed the normal powdered diet (control, *n* = 11). Observers remained anesthetized for 1–2 min after removal from isofluorane. During this recovery period they were placed in a standard cage and on a heated plate to keep them warm. They were then returned to their home-cage where they were allowed 2 h access to plain powdered food (∼2 g per mouse). On day 5, observers in each of the three groups were tested for their preference between cumin-scented and cocoa-scented food.

#### Training procedure 2—simulated social transfer of food preference

Preliminary behavioral experiments were conducted in order to establish the naïve preference in this paradigm between a number of different combinations of odor pairs. From these tests ginger was chosen as the training odor and coriander as an alternative (untrained) one because there was a stable apparently innate preference for coriander over ginger. For the task, observer mice (*n* = 37) were anesthetized (as in Training Procedure 1) and then transferred from the mask carrying the isofluorane and O_2_ anesthetic gas to one carrying the anesthetic gas with an odor introduced. Odors were added to the anesthetic gas by first drawing off a sample of the odor from an odor bag into a 50 ml syringe, then delivering this into the anesthetic gas using a syringe pump (Harvard Apparatus, UK—model 22). Three groups of mice were used: group 1 (Ginger + CS_2_, *n* = 14) received 30 ml/min ginger food odor with 15 ml/min CS_2_, group 2 (Ginger, *n* = 10) received 30 ml/min ginger food odor, and group 3 (CS_2_, *N* = 13) received 15 ml/min CS_2_. These odors were mixed with 120 ml/min of the anesthetic gas, and any shortfall beneath 150 ml/min was made up with N_2_ gas. Odor exposure was for 1 min. After this, they were placed in a heated cage to recover from anesthesia and then returned to their home-cage where they were allowed to feed for 2 h (as in Training Procedure 1). On day 5, observers in each of the three groups were tested for their preference between ginger-scented and coriander-scented food.

#### Food preference testing

Observer mice were tested individually under red light for food preference. On day 5 of restricted feeding, each mouse was placed in the center of the test arena in which there were two food cups in diagonally opposite corners (Figure 1), each containing a different scented food. Mice were left undisturbed for 30 min, monitored and recorded via an overhead video camera. Tests were conducted during the 2 h period when the mice had been fed on the preceding days of restricted feeding. At the end of the test the food remaining in each cup, and the amount displaced into the outer dish, was weighed to determine the amount of each food consumed.

For each food type the amount consumed in the test was expressed as a percentage of the total food consumption during the test. Training Procedure 1: Data were divided in to three groups depending on the food the demonstrator had eaten, cocoa-trained, cumin-trained and plain-trained. To establish whether there was a training related food preference, a Kruskall Wallis test followed by Dunn’s multiple comparisons test was used to compare the cocoa-scented food consumption.

### *In vivo* neurochemistry

#### Preparation for in vivo microdialysis sampling

**Figure 2:**
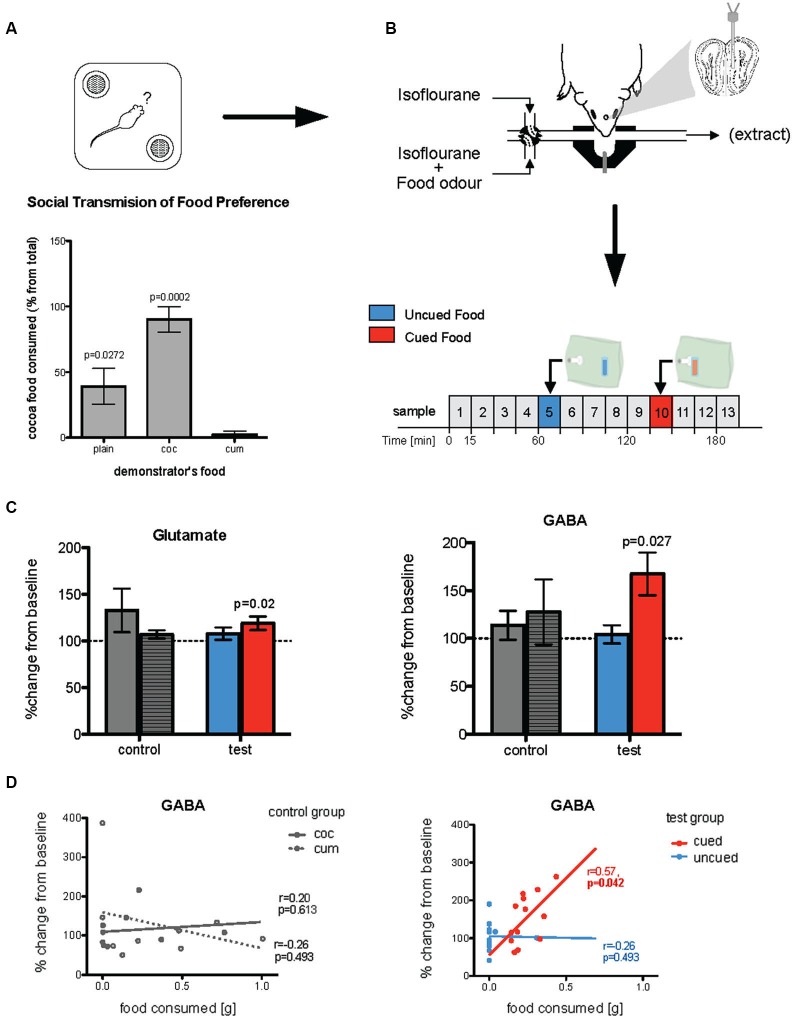
**Changes in *in vivo* glutamate and GABA following olfactory learning**. **(A)** Food preference test of observer mice where they were given a choice between cocoa- or cumin-scented foods, 24 h after exposure to the demonstrator’s breath when both observer and demonstrator were anesthetized (Training procedure 1). Histograms show relative consumption of cocoa-scented food (% from total intake). Relative consumption of the two foods was significantly different across the groups (*p* = 0.0007). While plain food-trained mice (controls, whose demonstrator had been given normal plain food) showed no significant preference for either food, cocoa-trained mice ate almost exclusively cocoa-scented food and cumin-trained animals ate nearly none (*p*-values given in the Figure are relative to cumin-trained mice). **(B)** Immediately after being tested for food preference, mice were used for microdialysis experiment. A microdialysis probe was placed in the posteromedial region of the olfactory bulb and 15 min microdialysis samples (25 µl) were taken. Odors were presented, via an odor containing bag, on samples 5 and 10. **(C)** In test mice (both cocoa- and cumin-trained animals) the concentrations of both glutamate and GABA sampled during exposure to the cued food odor were significantly increased relative to baseline (dotted line), while there was no such increase when exposed to the uncued odor. In the control (plain-trained) mice on the other hand there were no differences in concentrations of either transmitter during exposure to the two odors. **(D)** For test mice, the amount of cued food consumed during the preference test was positively correlated with concentrations of GABA collected during exposure to the cued but not uncued odor during microdialysis sampling (right panel). In the control mice, there was no such relationship between release of either transmitter during exposure to a food odor, and the quantity of that food consumed in the preference test (left panel).

Animals included in the microdialysis study were those used in the behavioral study under Training Procedure 1. Immediately after completion of the food preference test (Figure 2A), mice were anesthetized with an i.p. injection (0.1 ml/g body weight) of Avertin (5 g 1,2,2,2 -tribromoethanol, 3 ml 2-methylbutan-2-ol, 20 ml ethanol, 222 ml saline). Animals were then placed in a stereotaxic frame (Kopf Instruments, California) where they were fixed using ear bars and a bite bar. Anesthesia was maintained with 1.5% isofluorane in oxygen (150–200 ml/min) delivered through a diffuser (Univentor Ltd, Malta) through a mask over the nose. Body temperature was maintained using a homeothermic heated blanket system and a rectal probe (Harvard Instruments, UK). A microdialysis probe (MAB 4.15.2 Cu, Microbiotech, Sweden) was inserted unilaterally into the external plexiform layer of the left olfactory bulb through an incision in the scalp and a small craniotomy (AP 3.9 mm, LR 5.25 mm, DV 5.25; Figure 2B). Coordinates were calculated from the bregma and dural surface (dorso-ventral coordinates) according the mouse brain atlas of Paxinos and Franklin (2001) and were sufficiently posterior to avoid any possibility of sampling from the AOB.

#### Sampling protocol and analysis

Dialysis probes were perfused with a Krebs-Ringer solution (pH 7.4, 138 mM NaCl, 1.5 mM CaCl_2_, 11 mM NaHCO_3_, 5 mM KCl, 1 mM MgCl_2_, 1 mM NaH_2_CO_3_) at 1.5 µl/min, using a syringe pump (CMA-10; CMA Microdialysis, Sweden), throughout the course of the experiment. Sampling commenced 90 min after probe implantation. Samples (25 µl) were collected at 15 min intervals (Figure 2B) into tubes containing 2 µl acetic acid, and frozen (−20°C) immediately for further analysis by high performance liquid chromatography (HPLC). During the 5th and 10th of these intervals, the normal air supply to the mask was switched from the normal gas supply (isofluorane in O_2_ and N_2_) to a balanced supply carrying a food odor introduced from an odor bag (see above), cumin in one interval, cocoa in the other. The order of presentation of the two food odors was randomized between animals, and independent of the food consumed by the demonstrator animal. The odor, carried in N_2_, was diffused through a hypodermic needle (25 G, 0.5 × 16 mm) into a gas supply carrying isofluorane in O_2_ using a syringe pump (Harvard Apparatus, UK—Model 33 Twin Syringe Pump).

At the end of the experiment, mice were killed by cervical dislocation, brains were removed and probe placement confirmed. Concentrations of the amino acids glutamate, gamma-aminobutyric acid (GABA) and aspartate were quantified by HPLC with fluorescence detection following derivatization with ophthaldialdehyde (Sigma) performed with an autosampler (Gilson 231), as previously described (Kendrick et al., 1996).

All data were collected using Chromeleon 6.5 software (Dionex, Sunnyvale, USA). Stable, baseline concentrations of extracellular amino acids were taken from the average of the two samples preceding the odor challenge. Changes from baseline (100%) in response to the odor challenges were analyzed using a Wilcoxon matched-pairs signed rank test. Correlation between learning and changes in neurotransmitter release was assessed using a Spearman correlation test. The amount of cued food eaten was correlated with the percentage change in glutamate and GABA release in response to the cued food odor.

### Electrophysiological recording

#### Preparation for recording

**Figure 3:**
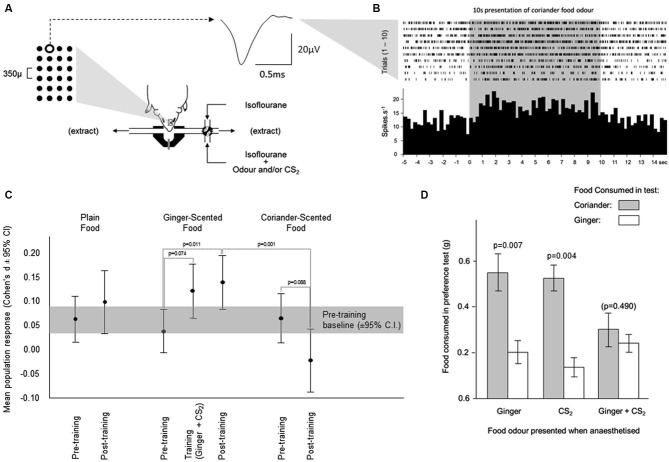
**Changes in neuronal activity during olfactory learning under anesthesia. (A)** Neuronal activity was sampled from neurons across a 6 × 4 array of tungsten microelectrodes (tip separation 350 µ) advanced into the olfactory bulb from the dorsal surface. Spikes from individual neurons were sorted from multineuronal activity at multiple channels across the array. The averaged waveform is shown for spikes generated by a single neuron at the position highlighted on the array. Odors were presented for 10 s, by switching the normal anesthetic gas supply with one carrying an odor. **(B)** The times of occurrence of spikes generated by the neuron depicted in **(A)** are shown during presentations of the coriander food odor. The histogram shows the average response across 10 presentations of this odor. **(C)** Each odor was presented 10 times before training, and 10 times after training. Training comprised 10 presentations of the ginger food odor combined with CS_2_. The response during training was elevated relative to the response to ginger alone before training. This enhanced response achieved significance after training when ginger was presented in the absence of CS_2_. The response to coriander fell from pre- to post-training. **(D)** In the pilot behavioral simulation of social transfer of odor preference, mice were anesthetized and presented with odors carried in the anesthetic gas; ginger, CS_2_, or ginger and CS_2_ combined. When tested for preference between coriander and ginger flavored foods, mice which had been exposed to the ginger odor or CS_2_ separately preferred coriander over ginger. In those exposed to ginger and CS_2_ combined, this preference was overcome, and the mice consumed similar amounts of both foods.

After initial induction of anesthesia (25% hypnorm, 25% hypnovel, i.p., 0.01 ml/g body weight), mice were placed on a heated blanket (33.4°C) in a stereotaxic frame, and their heads fixed with ear bars and a bite bar. Anesthesia was maintained with 1.5% isofluorane in air (30% Nitrogen, 70% Oxygen) delivered through a mask over the nose. Breathing was monitored using a thermistor located in the mask (see Figure 3A).

The left olfactory bulb was exposed through a dorsal craniotomy large enough to accommodate the electrode array (approx. 2 mm AP × 1.5 mm ML), and the dura retracted. Exposed tissue was bathed in sterile saline delivered at 33.4°C. A 6 × 4 electrode array (sharpened tungsten microelectrodes, 300–500 kΩ, tip separation 350 µ) was positioned at the surface of the bulb, and advanced until spontaneous extracellular neuronal activity (action potentials, “spikes”) were detected across a substantial portion of the array, ranging from 3–15 electrodes per mouse (average = 8 electrodes). The objective was to make recordings from the dorsal mitral cell layer across the majority of the main olfactory bulb. Once this condition was satisfied, the preparation was left to stabilize for a period of no less than 2 h. The placement of recording arrays was sufficiently posterior to avoid any possibility of recordings being made from the AOB.

#### Recording protocols and analysis

Neuronal activity was sampled using a 64 channel Plexon Multichannel Acquisition Processor (MAP, Plexon Inc., USA). The signal from each electrode was sampled at 30 kHz. Extracellularly recorded action potentials (spikes) were isolated from the continuous signal when this signal exceeded a negative triggering threshold set at an estimated 5× background (∼−25 µV). Experimental markers and ventilation were recorded using a Power1401 laboratory interface and Spike2 software (Cambridge Electronic Design Ltd., UK). This system was also used for experimental control.

An experimental trial comprised a pre-stimulus period of at least 10 s, during which ventilatory and neuronal activity were visually monitored for stability, followed by a 10 s odor presentation (see Figure 3B). Using the animals’ monitored ventilation, the onset of odor presentation was automated to a point mid-way through exhalation, odor onset occurring at the first such point after the 10 s stable pre-stimulus period. Odor delivery was as described above in neurochemistry methods (sampling), with the exception that the odors used here were ginger and coriander, or ginger paired with CS_2_ during the training phase. Neuronal activity sampled during inhalation and exhalation was considered separately in subsequent analyses.

Recordings were made during three experimental phases: (1) pre-training; (2) training; and (3) post-training. In the pre-training phase, mice were presented with the three food odors: normal, ginger and coriander. These were presented in blocks of 5 trials, in balanced order, a total of 10 trials per odor. Training comprised 10 trials in which the training odor (ginger) was presented in combination with CS_2_. The post-training phase replicated the pre-training phase, using the same presentation order as used in pre-training recordings. Throughout the recordings, an inter-trial interval of ≥5 min was used. For some of the animals, a final block of trials was conducted in which CS_2_ alone was presented. On completion of the electrophysiological recordings, animals received a lethal dose of pentobarbital (Dolethal, 0.5 ml i.p.).

Initial processing of the data to extract the times and waveforms of spikes in relation to stimulus and ventilatory information was performed using Spike2 software. For the majority of electrodes where spikes were detected, these represented the activities of multiple neurons. The activities of individual neurons were sorted from multineuronal activity using a machine learning algorithm applied to principal components extracted from the spike waveforms using principal components analysis, and waveform properties detected by curve fitting (Horton et al., 2007). This spike-sorting technique was realized in custom-written software running in MatLab. For each trial, the activity of each neuron was partitioned into data sampled during inhalation or during exhalation, and data sampled pre-stimulus presentation, or during stimulus presentation.

On the basis of data sampled during inhalation in the pre-stimulus period, the activity of each neuron was categorized as parametric or non-parametric. In each trial, the ratio of mean to standard deviation was calculated for the firing rate of each neuron. The average of this ratio was then calculated across the full set of trials for each stimulus condition. If this average was ≥2, the neuron was defined as “parametric” under the given condition, meaning that the distribution of the spikes across the time windows was considered normal. If the average was <2, the neuron was defined as “non-parametric”. This property was taken into account in subsequent analyses.

Other studies mainly report weaker neuronal responses to sensory stimuli in anesthetized animals when compared to awake animals (see Brown and Horn, 1994), and rely on cumulative change in activity across multiple recordings (e.g., McLennan and Horn, 1992), although one study on the mouse olfactory bulb has reported stronger responses to odor stimuli under ketamine anesthesia (Rinberg et al., 2006). Here we have adopted a similar approach by using change in activity across multiple neurons, but also address the issue of weak single neuron responses by employing Cohen’s D as a measure of response strength. Using this approach, single neurons were classified as weakly, moderately or strongly responsive.

Neuronal responses were quantified using the squared log ratio of the firing rate during stimulus presentation relative to the firing rate before. This was calculated for each neuron across the 10 trials (considered as technical replicates). Only the neurons for which data were obtained under the full series of seven conditions (plain, ginger and coriander before and after training, and training with ginger and CS_2_) were considered in analyses. Initially focusing on the neurons identified as parametric (see above), two-way repeated measures ANOVA was applied, with odor as the within group factor and subject as the between group factor.

## Results

### Neurotransmitter changes in response to learned odors

Prior to preparation for neurochemical analysis by *in vivo* microdialysis, mice were anesthetized and trained by social transfer of food preference from an anesthetized conspecific (Training Procedure 1, Figure 1). Twenty-four hours after recovery from anesthetic, they were tested for food preference (Figure 2A). The total amount of food consumed in the preference test by observer mice in each group was not significantly influenced by the food used to load the demonstrator (0.47 sg plain-trained, 0.27 g cocoa-trained and 0.25 g cumin-trained—*p* > 0.05). However, the relative consumption of each food by the observers in the preference test varied significantly according to the odor presented in the demonstrator’s exhaled breath when both animals were anesthetized (Kruskal-Wallis, $\chi_{2}^{2}=142.67$, *p* = 0.0007). The trained observers consumed more of the training food than the alternative food, cocoa-trained groups ate significantly more of the cocoa-scented food than the cumin-trained group (Dunn’s Multiple Comparison Test, *p* < 0.001). Mice in the control group (exposed to the breath of demonstrators fed with plain food) showed no significant preference for either scented food, although they ate significantly more cocoa-scented food than cumin-trained animals (Dunn’s Multiple Comparison Test, *p* < 0.05). On completion of these procedures, animals were again anesthetized and prepared for *in vivo* microdialysis (Figure 2B).

The full microdialysis sampling protocol was successfully completed in 13 trained and 9 control mice. Four trained and two control animals were either excluded either because the full sampling protocol could not be completed due to technical problems or, in the trained group, did not show clear evidence of a learned preference. Figure 2B shows the typical medial posterior olfactory bulb placements of microdialysis probes. Glutamate and GABA concentrations were measured in 15 min microdialysis samples collected during both baseline conditions and exposure to the different odors (i.e., cocoa-scented and cumin-scented food odors; see protocol in Figure 2B). Changes in transmitter concentrations in samples taken during odor presentations were calculated relative to the two preceding 15 min baseline sample periods.

In mice trained by exposure to demonstrators that had eaten a scented food (cumin or cocoa), glutamate and GABA concentrations were only significantly increased in microdialysis samples taken during presentation of these same odors (Wilcoxon test: Glutamate *p* = 0.0237; GABA *p* = 0.0266—Figure 2C). There was no significant change in the ratio of glutamate to GABA during presentation of the cued odor (*p* > 0.1) although the magnitude of evoked GABA release tended to be higher than that of glutamate. There were no significant changes in the concentrations of either glutamate or GABA, or in the glutamate/GABA ratio in samples collected during exposure to the alternative control odor not present on the demonstrator’s breath. There were no changes in glutamate or GABA concentrations during presentation of either odor to control mice which had been exposed to demonstrator mice that had eaten only plain unscented food (Figure 2C).

The increased concentrations of GABA during presentation of the cued food were significantly positively correlated with the amount of that food consumed in the preference test (Spearman rank test: *r* = 0.57, *p* = 0.042; Figure 2D). There was no significant correlation between either glutamate or GABA concentrations and the total amount of food consumed in the preference test. Furthermore, in the untrained controls the combined consumption of both foods in the preference test was not significantly correlated with release of either transmitter (Figure 2D).

### Multiarray electrophysiological changes in response to learned odors

#### Behavior

In the electrophysiological studies ginger was used as the training odor and coriander as the alternative test odor. These were delivered from odor bags rather than anesthetized demonstrators (see Training Procedure 2). Mice in three groups were each exposed to different odors while anesthetized: (1) ginger + food odor; (2) CS_2_; and (3) ginger + food odor paired with CS_2_. While the total amount of food consumed in the preference test did not vary across the three groups the relative consumption of coriander-scented food and ginger-scented food in the preference test reflected odor exposure under anesthesia. In both control groups, mice exposed under anesthesia to either ginger or CS_2_, food consumption indicated a clear preference for coriander over ginger (Figure 3D; paired *t*-tests, Ginger: *t*_9_ = 3.46, *p* = 0.007 and CS_2_: *t*_12_ = 3.61, *p* = 0.004). Mice which had been exposed under anesthesia to Ginger and CS_2_ combined showed no such preference since they consumed similar quantities of both foods (Figure 3D). A comparison of the Ginger and CS_2_ alone groups combined with the Ginger +CS_2_ group also showed a significant difference in terms of the relative consumption of ginger-scented food (*t*_33_ = 3.295, *p* = 0.002). Thus, in these mice the innate preference for coriander over ginger seen in controls was overcome.

#### Electrophysiological recordings

Electrophysiological recordings were made from a separate cohort of mice to those used in the behavioral tests described above. The mice were anesthetized throughout all electrophysiological recording procedures and their breathing pattern was not influenced differentially by the odors used. While the first breathing cycle (inhale and exhale) after odor presentation was significantly faster than the average pre-stimulus breathing rate, this did not vary across the different odors and returned to normal in the second and subsequent breathing cycles during stimulus presentation.

In seven mice, neuronal activity was detected at a total of 50 electrodes (3–15 active channels per animal). From these spike trains, the activity of 260 individual neurons was discriminated by spike sorting (Horton et al., 2007).

For each experimental condition, neurons were identified as parametric or non-parametric on the basis of their background activity (prestimulus activity) during inhalation. Neurons classified in this way did not necessarily remain in the same category across all conditions.

Analyses of odor-evoked changes in neuronal activity were initially performed on activity sampled during inhalation amongst neurons classified as parametric. Activity recorded during odor presentation was compared to that in the pre-stimulus period across presentations of each odor.

#### Changes in multiunit activity

In the pre-training period (i.e., before presentation of the training odor combined with CS_2_), a similar increase in multiunit activity was evoked by presentation of each food odor in the parametric neurons (mean ± sem respectively plain 5.2% ± 2.4, ginger 3.5% ± 2.1; coriander 5.4% ± 2.7—Figure 3C). During training, the response to the ginger food odor combined with CS_2_ showed a trend towards being greater than that evoked by presentation of the ginger food odor alone (paired *t*-test, *t*_84_ = 1.81, *p* = 0.074, 10.4% ± 2.7—Figure 3C). After training, the response to the ginger food odor alone (13.03% ± 2.7—Figure 3C) was significantly greater (*t*-test vs. ginger pretraining, *t*_84_ = 2.58, *p* = 0.011) than that evoked by presentation of this odor combined with CS_2_ during training, and significantly greater than the responses to the coriander food odor (paired *t*-test, *t*_84_ = 3.47, *p* = 0.001), which was not the case before training (paired *t*-test *p* = 0.577). For all but one of the mice, there was a consistent increase in the size of the response to the ginger odor in multiunit activity amongst parametric neurons; in the remaining mouse the size of this response did not increase. Significant variability between mice was related to the size of the training-related increase in the response to the ginger food odor. After completion of the post-training tests for responsiveness to the three food odors, some animals (*n* = 3) were also tested with CS_2_ alone. The multiunit response to this odor was similar to the pre-training responses to food odors confirming that CS_2_ odor alone did not evoke a differential response compared to the other odors used.

In the multiunit activity sampled during exhalation from parametric neurons, there was a significantly larger increase in activity in response to presentation of the ginger food odor combined with CS_2_ during training than in response to any of the three food odors in the pre-training recordings. However, none of these responses to the food odors was altered after training.

The general profile of responsiveness in the multiunit activity amongst the non-parametric neurons was similar to that seen in the parametric ones (data not shown).

#### Changes in single neuron activity

The responses of single neurons in these recordings were generally weak, and few reached significance using conventional methods (e.g., *t*-tests). Using Cohen’s D as a measure of response strength yielded a larger sample of responsive neurons, thereby improving the power of subsequent analyses investigating the changing profiles of neuronal responsiveness to the different odors.

The proportion of single neurons showing increased activity in response to the ginger food odor after training showed a trend towards significance (29/85, 34% before and 39/85, 46%—McNemar test, *p* = 0.123) as well as during the training period when ginger was presented with CS_2_ (40/85, 47%, *p* = 0.1). This increase was largely confined to neurons producing excitatory responses (increase in firing rate) with the proportion of neurons producing inhibitory responses remaining stable. The proportion of neurons responding to plain food odor did increase significantly from pre-training (33/85, 39%) to post-training (47/85, 55%, *p* = 0.005). Again this increase was confined to the neurons producing excitatory responses, but here was also accompanied by a decrease in the proportion of neurons producing inhibitory responses. The proportion OF neurons responding to the coriander food odor increased slightly, but not significantly, between the pre-training (34/85, 40%) and post-training (41/85, 48%, *p* = 0.349) periods, although that of neurons with an excitatory response to coriander declined, but not significantly, from pre-training (25/85, 29%) to post-training (19/85, 22%, *p* = 0.391). Among the 85 recorded individual neurons, 6 changed their response to coriander from excitatory to inhibitory after training, whereas 13 changed their response to ginger from inhibitory to excitatory. This difference in the pre vs. post-training pattern of response to the two odors was significant (McNemar test, *df* = 84,*p* = 0.016).

#### Localization of learning-evoked electrophysiological changes

**Figure 4:**
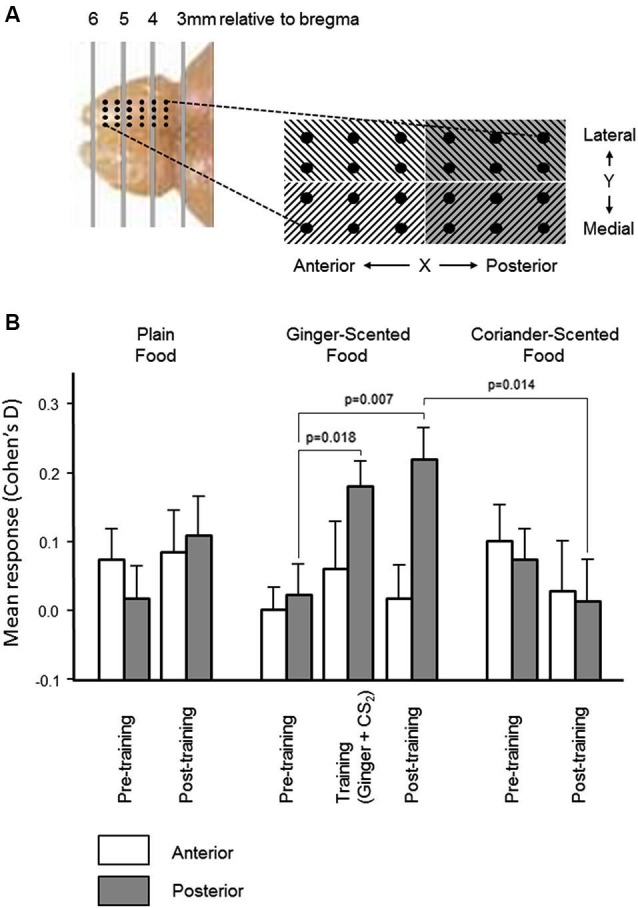
**Spatial distribution of olfactory bulb neural responses during learning.** **(A)** In order to establish the spatial distribution of olfactory bulb mitral cell responses and learning-related changes, the microelectrode array was partitioned mediolaterally and anteroposteriorly (photograph and schematic illustrate the localization of the 24-electrode arrays). There was no significant variation in multiunit responses, or in learning-related changes, across the mediolateral dimension of the array. Histograms in **(B)** show that the increase (mean ± sem) in the size of the multiunit responses to the combined presentation of ginger and CS_2_, and the learning-related increase in the size of the multineuron response to ginger was restricted to the posterior half of the array.

Preliminary analyses of the spatial distribution of responsiveness and learning-related effects were made by comparing effects across the X (anteroposterior, 6 columns of electrodes) and Y (mediolateral, 4 rows) dimensions of the electrode array (see Figure 4).

In the pre-training period, there were no significant differences in either dimension in the change in multiunit activity evoked by presentation of any of the three odors (ginger, coriander, or plain food). In the post-training period, there was also no significant difference in the distribution of learning-related change in responsiveness in the Y dimension of the array (i.e., medial vs. lateral). However, the increased multiunit response to ginger following training exposure to ginger combined with CS_2_ (see Figure 3) was restricted to the posterior part of the array (Figure 4). Only in this part of the array were the changes in multiunit activity evoked by presentation of ginger and CS_2_ during training, and by ginger alone after training, significantly greater than the multiunit response to ginger before training (paired *t*-tests, *t*_30_ = 2.5, *p* = 0.018 and *t*_30_ = 2.89, *p* = 0.007 respectively). Also, only in the posterior part of the array was the multiunit response to ginger significantly greater than that to coriander after training (*t*_30_ = 2.62, *p* = 0.014). Furthermore, after training there were significantly more excitatory than inhibitory responses to ginger in the posterior part of the array (23% before vs. 37% after, *p* < 0.05). There was no change in responsiveness to either of the other stimuli. In the anterior part of the array, there was no training-related change in the size of the response to any stimulus, and no significant difference between excitatory and inhibitory responses either before or after training.

## Discussion

Overall our results have provided both neurochemical and electrophysiological evidence *in vivo* that similar plasticity changes occur in mouse olfactory bulb encoding associated with learning under anesthesia as previously reported in olfactory learning contexts in conscious animals (Wilson et al., 1987; Kendrick et al., 1992, 1997; Brennan et al., 1998). This provides yet further evidence that the mammalian brain can undergo plasticity changes and learning in unconscious as in conscious states and also demonstrates similarities in altered olfactory bulb encoding associated with olfactory learning in both cases. Our findings also establish this anesthetized learning model as potentially useful for more detailed investigation of the precise neural encoding changes associated with learning which can be more difficult to achieve using awake, behaving animals.

Our present results provide support for our previous findings that learning in the social transfer of food preference paradigm can occur under anesthesia (Burne et al., 2010). Our previous study showed however that food preference transferred in this way is influenced by different types of anesthetic agents which may depend upon which transmitter receptors are targeted by them (Burne et al., 2010). Specifically, those acting by blocking NMDA receptor activity (e.g., Vetalar—Ketamine hydrochloride) appear to prevent acquisition of preference for a novel food, whereas a GABA receptor agonist (Sagatal), produce an aversion rather than a preference for the odor presented during anesthesia. Indeed, a previous study has reported increased responsivity to odors in olfactory bulb mitral cells under ketamine anesthesia in mice, compared to conscious recordings (Rinberg et al., 2006), possibly suggesting reduced selectivity and increased noise in mitral cell networks might contribute to its blockade of learning effects. In the present work, we therefore used isofluorane which is a membrane stabilizer that does not act specifically on receptor mechanisms, and leaves cortical neurons responsive to synaptic input (Orth et al., 2006).

Our observed *in vivo* increases in release of both glutamate and GABA in the olfactory bulb in response to learned odors in anesthetized mice is similar to that observed in conscious sheep (Kendrick et al., 1992, 1997) and mice (Brennan et al., 1995) in other olfactory learning paradigms. Importantly, in the current study strength of learning, as indexed by the amount of cued odor food eaten, was positively correlated with the magnitude of GABA concentration changes. Overall this provides further support for a common mechanism whereby learned odors evoke both a greater excitatory mitral cell (glutamate) and greater inhibitory granule cell (GABA) responses in the olfactory bulb. Unlike studies in conscious mice (Brennan et al., 1995) and sheep (Kendrick et al., 1992) there was no significant increase in the ratio of glutamate to GABA associated with olfactory learning in the current study, although there was a trend in this direction with the change in learned odor-evoked GABA release being slightly greater than for glutamate. This might reflect an impact of anesthesia in weakening learning-induced facilitation of mitral to granule cell communication via enhanced dendrodendritic synaptic sensitivity and/or the different learning paradigm used compared with other studies.

The findings from multi-array electrophysiological recordings provide further evidence that learned associations with complex odorants result in an altered response profile across an extensive population of mitral cell neurons. Before training by exposure to ginger and CS_2_ together, responses (both in multiunit and single neuron activity) to each food odor (plain, ginger, coriander) were similar, and also similar to those for CS_2_ presented alone, and no differential effects of odors were seen on breathing rate. Thus, CS_2_ itself did not evoke greater changes than other odors and its presentation was only accompanied by an elevated response when it was paired with the ginger food odor. After training, increased responsiveness was maintained only for the ginger food odor and this elevated response to the training odor was rapid, occurring shortly after its combination with CS_2_. In agreement with single neuron recordings from the olfactory bulb of the conscious maternal sheep which had learned to recognize the odors of their lamb (Kendrick et al., 1992), the cued odor primarily evoked an increase in the proportion of neurons exhibiting excitatory responses. While we cannot rule out the possibility that some of the neurons recorded by the array were inhibitory granule cells, the electrodes are likely to have been primarily mitral cells. This assumption is based on (a) the depth of penetration of the microelectrode array; and (b) the large signal to noise ratio of the spikes detected which is characteristic of the mitral cell rather than the granule cell layer; and (c) since granule cells do not have an axon they are less likely to generate “spikes” which can be recorded extracellularly. As such the activity detected represents the output from the granule cell layer, where the learning processes are thought to take place in the network between inhibitory granule cells and mitral cell dendrites (Brennan and Keverne, 1997; Brennan and Kendrick, 2006; Sanchez-Andrade and Kendrick, 2010). Thus it would appear likely that learning primarily increases responses of glutamatergic mitral cell neurons to the learned odor, which is consistent with increased *in vivo* glutamate release.

Despite evidence for enhanced release of GABA in the olfactory bulb during exposure to the learned odor this does not appear to be associated with extensive reductions in mitral cell responses to learned odors in the olfactory bulb of the anesthetized mouse, in the current study, or in the conscious sheep (Kendrick et al., 1992). This contrasts with conscious recordings from the mouse AOB in the pregnancy block model where mating produced a reduction in mitral cell responses to the learned odors from the urine of a male (Binns and Brennan, 2005). However, this may reflect the fact that in the latter model it is proposed that pregnancy block does not occur in response to the familiar male because his odors fail to induce increases in mitral cell responses which would promote changes in the hypothalamo-pituitary axis and subsequent pregnancy termination (see Brennan and Kendrick, 2006). For the main olfactory system on the other hand it is assumed that learned odors with positive associations can more effectively promote behavioral and endocrine changes through an enhanced response in mitral cells having a greater impact on the relevant downstream projection region (see Brennan and Kendrick, 2006; Sanchez-Andrade and Kendrick, 2010). Possibly the parallel enhancement of inhibitory GABA release in this case is more important for helping to minimize interference from other, potentially competing odors, by reducing responses of other adjacent mitral cells. Indeed, the finding in the current study of a trend towards an increased proportion of mitral cells exhibiting reduced responses to the uncued odor supports such a possibility, and similar evidence for reduced mitral cell responses to other odors has also been observed in the sheep olfactory bulb (Kendrick et al., 1992). In such a system, increased lateral inhibition through elevated GABA release could also contribute to facilitated responses to the cued odor. Interestingly, similar evidence for increased inhibitory responses to unlearned stimuli has been reported in the chick brain following visual imprinting (Nicol et al., 1995; also Nicol et al., submitted). Enhanced inhibition of mitral cells via granule cells is associated with improved discrimination between odors (Abraham et al., 2010), and thus learning-associated changes in odor-evoked GABA release may reflect an improved ability to discriminate learned odors from other potentially competing ones.

In the context of the current study the dual influence of training in enhancing responses to the cued (ginger) odor but reducing them to an uncued (coriander) one suggests that the changes in responsiveness to ginger food odor were not simply due to sensitization following exposure, as similar exposure to each food odor induced contrasting results. Further, short-term changes in responsiveness were not apparent in pre-training exposures to any food odor and no significant changes were observed in responses across the 10 trials with each odor.

The complex odors used in our study influenced the activity of a large proportion of mitral cells recorded by the recording arrays in line with a recent study showing dense representation of natural odorants in the mouse olfactory bulb (Vincis et al., 2012). On the other hand learning associated changes were localized to mitral cell populations in the dorsal posterior rather than anterior part of the olfactory bulb suggesting a degree of spatial localization, at least in terms of neural plasticity changes. *In vivo* neurochemical changes associated with learning were also found in posterior regions of the olfactory bulb although we did not investigate whether they were absent in more anterior regions. Possibly our electrophysiological findings may reflect differential localization of processing for innate compared with learned odors reported previously in the mouse olfactory bulb (Kobayakawa et al., 2007). This is in accordance with findings that the necklace glomeruli receiving input from the specialized guanylyl cyclase GC-D sensory neurons in the posterior region of the olfactory bulb responds to social signals like CS_2_ and mediate social transmission of food preference in mice (Munger et al., 2010). Perhaps more anterior populations of dorsal mitral cells are innately tuned to other classes of key biological odorants which exhibit less plasticity in response to new learned associations. Clearly such a possibility requires further investigation.

A limitation of our study is that the large differences in scale for olfactory bulb activity changes made using microdialysis sampling and recording microelectrodes make it difficult to draw anything other than tentative conclusions linking neurotransmitter changes with altered mitral cell responses. It is also important to emphasize the temporal differences in the two different sampling approaches as well as the fact that in the microdialysis experiment changes were sampled in the olfactory bulb 24 h after learning whereas for the recording experiments changes were restricted to a relatively short period before, during and immediately after learning. Thus the pattern of neurochemical changes measured would therefore have reflected post-consolidation learning whereas the electrophysiological changes are associated with initial acquisition of learning and may have been further modified post- consolidation. However, other studies of consolidation in visual recognition memory suggest that neurons recruited during in acquisition are likely to be those maintained post-consolidation (Horn et al., 2001; Jackson et al., 2008). Recordings were also restricted to the dorsal mitral cell layer and other patterns of response might have occurred in lateral and ventral layers.

Our findings provide further evidence that the mammalian brain is capable of supporting some forms of learning under anesthesia, although to the best of our knowledge this is the first study to show detailed neurochemical and neurophysiological plasticity changes occurring in a primary sensory processing region. A converging body of evidence has also implicated sleep as being crucial in memory formation (Ellenbogen et al., 2006; Arzi et al., 2012), and specifically in memory consolidation (Walker and Stickgold, 2004; Rasch et al., 2007; Jackson et al., 2008), and thus learning-related changes during unconscious states would appear to be both important and complimentary to those occurring during conscious ones. While we have observed broadly similar patterns of neural plasticity changes in the olfactory bulb under anesthesia to those known to occur under conditions of conscious learning more detailed comparisons of learning using the same paradigm under conscious and unconscious conditions are required before we can establish whether certain differences exist. In particular factors such as the duration and specificity of memory and neural plasticity changes need further investigation. A further interesting question is whether the established role of neurogenesis in some learning paradigms involving both the hippocampus and olfactory bulb (Lazarini and Lledo, 2011) can also occur under anesthesia or other unconscious states.

## Conclusions

Overall we have provided the first in vivo neurochemical and neurophysiological evidence for olfactory learning under anesthesia in mice being supported by broadly similar changes in olfactory bulb encoding as shown previously in conscious paradigms and involving potentiated glutamate and GABA release from mitral and granule cell synapses. Observed changes support the presence of dense, but also spatially restricted representation of changes with the olfactory bulb associated with learned associations with complex odorants. The pattern of changes found reveal enhanced excitatory responses in mitral cell populations but also corresponding reductions in responses in those tuned to other, potentially interfering odors.

## Author contributions

Alister U. Nicol, Gabriela Sanchez-Andrade, and Keith M. Kendrick conceived and designed the study; Alister U. Nicol, Gabriela Sanchez-Andrade and Paloma Collado carried out the experimental work; Alister U. Nicol, Gabriela Sanchez-Andrade, Anne Segonds-Pichon and Keith M. Kendrick analyzed and interpreted the data; Alister U. Nicol, Gabriela Sanchez-Andrade, Anne Segonds-Pichon, Paloma Collado and Keith M. Kendrick drafted and approved the final version of the paper.

## Conflict of interest statement

The authors declare that the research was conducted in the absence of any commercial or financial relationships that could be construed as a potential conflict of interest.

## Footnotes

^1^http://www.legislation.gov.uk/ukpga/1986/14/enacted
